# Cerebellar Gray Matter Volume in Tinnitus

**DOI:** 10.3389/fnins.2022.862873

**Published:** 2022-04-29

**Authors:** Lilian M. Mennink, Elouise A. Koops, Dave R. M. Langers, Marlien W. Aalbers, J. Marc C. van Dijk, Pim van Dijk

**Affiliations:** ^1^Department of Neurosurgery, University Medical Center Groningen, University of Groningen, Groningen, Netherlands; ^2^Department of Otorhinolaryngology, Head and Neck Surgery, University Medical Center Groningen, University of Groningen, Groningen, Netherlands; ^3^Research School of Behavioral and Cognitive Neurosciences, University Medical Center Groningen, University of Groningen, Groningen, Netherlands; ^4^Department of Bio-informatics, Hanze University of Applied Sciences Groningen, Groningen, Netherlands

**Keywords:** tinnitus, cerebellum, flocculus, paraflocculus, hearing loss, gray matter volume

## Abstract

Tinnitus is the perception of sound without an external source. The flocculus (FL) and paraflocculus (PFL), which are small lobules of the cerebellum, have recently been implicated in its pathophysiology. In a previous study, the volume of the (P)FL-complex correlated with tinnitus severity in patients that had undergone cerebellopontine angle (CPA) tumor removal. In this study, the relation between tinnitus and gray matter volume (GMV) of the (P)FL-complex, GMV of the other cerebellar lobules and GMV of the cerebellar nuclei is investigated in otherwise healthy participants. Data was processed using the SUIT toolbox, which is dedicated to analysis of imaging data of the human cerebellum. GMV of all cerebellar lobules and nuclei were similar between tinnitus and non-tinnitus participants. Moreover, no relation was present between tinnitus severity, as measured by the Tinnitus Handicap Inventory, and (P)FL-complex GMV, tonsil GMV, or total cerebellar cortical GMV. These results suggest that in otherwise healthy participants, in contrast to participants after CPA tumor removal, no relation between the GMV of neither the (P)FL-complex nor other cerebellar lobules and tinnitus presence and severity exists. These findings indicate that a relation only exists when the (P)FL-complex is damaged, for instance by a CPA tumor. Alternatively, it is possible that differences in (P)FL-complex GMVs are too small to detect with a voxel-based morphometry study. Therefore, the role of the (P)FL-complex in tinnitus remains to be further studied.

## Introduction

Subjective tinnitus is the perception of sound without the presence of an external acoustic stimulus. It is present in 10–15% of the general population, and the prevalence increases with age (Baguley et al., 2013; McCormack et al., 2016). Three to thirty percent of cases report their tinnitus to be bothersome (McCormack et al., 2016). Severe tinnitus has been associated with frustration, irritability, anxiety, depression, insomnia and concentration difficulties, and therefore negatively affects the quality of life (Eggermont and Roberts, 2004; Langguth et al., 2013). Up to this date, no curative treatment exists.

The pathophysiology of tinnitus is still not completely elucidated. Since the severing of the auditory nerve does not diminish tinnitus, the generation of tinnitus probably involves a central mechanism (Jackson, 1985). Currently, tinnitus is considered a maladaptive neuroplastic response to sensory deprivation (Baguley et al., 2013). This maladaptive response has been associated with aberrant neuronal firing. Reported changes in neural activity are increases in spontaneous firing rates and temporal neural synchrony (Shore et al., 2016). Neural plasticity processes, such as changes in (inhibitory) neurotransmission or tonotopic map reorganization have been reported as potential mechanisms underlying the altered neural activity (Eggermont and Roberts, 2004; Wang et al., 2011; Baguley et al., 2013; Knipper et al., 2013; Shore et al., 2016; Koops et al., 2020b).

Although most research on tinnitus is focused on the classical auditory pathway, other brain structures, such as the anterior insula, anterior cingulate, hippocampus and cerebellum, have gained interest in the last few decades (Langguth et al., 2013). Within the cerebellum, the role of the paraflocculus (PFL) has been recognized. The flocculus (FL) and PFL are small lobules of the cerebellum involved in the vestibular function. Recently, there has been increasing evidence that the PFL is involved in tinnitus. Animals with behavioral signs of tinnitus after monoaural noise exposure showed increased activity in the PFL ipsilateral to the trauma ear in a manganese-enhanced MRI study (Brozoski et al., 2007). Moreover, in salicylate induced tinnitus in rats, both paraflocculi showed enhanced activity and increased functional connectivity with the auditory cortex in an fMRI study (Chen et al., 2015). Pharmacological activation and blocking of glutamatergic PFL-receptors exacerbated and partially attenuated tinnitus, respectively (Bauer et al., 2013b). Furthermore, pharmacological or surgical inactivation of the PFL eliminated behavioral signs of tinnitus and increased the spontaneous firing rate in the contralateral inferior colliculus (Bauer et al., 2013a; Vogler et al., 2016). In summary, PFL-activity is increased in animals with tinnitus, and tinnitus can be modulated by intervening directly in the PFL. This modulation may occur via an auditory feedback loop between the auditory cortex and the PFL. This feedback loop may become more activated when tinnitus exists, and intervention within this feedback loop may modulate tinnitus (Mennink et al., 2020).

**FIGURE 1:**
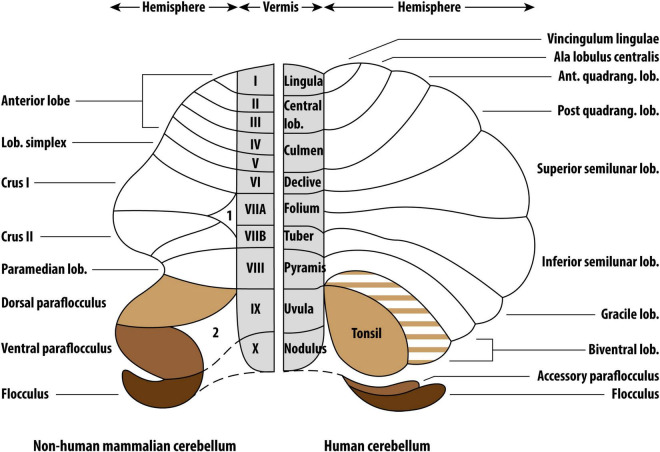
Comparative anatomy of the non-human mammalian and human cerebelli. The non-human mammalian FL, ventral PFL and dorsal PFL are the parallels of the human FL, accessory PFL and cerebellar tonsil, respectively. Figure reproduced from Mennink et al. (2020) (CC BY 4.0).

The PFL may not only influence tinnitus in animals, but also in humans. In humans, both the FL and PFL are located in the cerebellopontine angle (CPA). Although the cerebellum is highly comparable within mammals, translation of animal research to humans is not straightforward with regard to the PFL. In animals, the PFL consists of the dorsal and ventral PFL. It appears that the ventral PFL in animals corresponds to the human accessory PFL, but this is still a matter of debate (Figure 1). Likewise, the dorsal PFL in animals seems to correspond to the human cerebellar tonsil, and some reports even include the biventral lobule (Voogd and Ruigrok, 2012; Mennink et al., 2020). Rarely, animal studies on auditory function or tinnitus distinguish between the ventral and dorsal PFL. In humans, neuroanatomical tracing studies have not focused on the auditory function of the FL, accessory PFL, and cerebellar tonsil. Therefore, it is not entirely clear whether the previously described results of animal studies correspond to the human accessory PFL or cerebellar tonsil. When the PFL in humans is mentioned in this study, the accessory PFL is meant.

In humans, the role of the FL and PFL has been studied in only two studies. One of those is our previous study, in which we evaluated the role of the FL and PFL in patients after the surgical removal of a CPA-tumor (Mennink et al., 2018). In this population, 71% of patients had tinnitus postoperatively. The FL and PFL were measured together [(P)FL-complex], since it is not possible to delineate the individual PFL on an MRI-scan. There was no difference in (P)FL-complex volume between patients with and without tinnitus after CPA-tumor surgery. However, there was a positive correlation between the (P)FL-complex volume and the Tinnitus Functional Index (TFI) score, which indicates the severity and nuisance of the tinnitus. Patients with a bigger (P)FL-complex had more severe tinnitus and vice versa. Although this relationship was more pronounced on the side where the CPA tumor was removed, it was also present on the healthy side (Mennink et al., 2018). This endorses the hypothesis that the (P)FL -complex influences tinnitus not only in animals, but in humans as well. Sahin et al. (2020) found no differences in the GMV of the flocculonodular lobe between tinnitus and non-tinnitus participants that were otherwise healthy, but did not assess the relation between GMV and tinnitus severity. As such, the current study investigated whether the positive relation between tinnitus handicap and (P)FL volume is also present in a more general population of people with tinnitus, who did not undergo CPA-surgery. Moreover, Mennink et al. (2018) did not have audiometric data and could not correct for total brain volume. Therefore, the current study additionally aimed to evaluate the relation between the (P)FL-complex gray matter volume (GMV) and tinnitus in otherwise healthy participants with incorporation of audiometric and brain volume data. Last, since it is interesting to know whether potential effects are specific for the (P)FL-complex, or are rather based on other cerebellar effects, all cerebellar lobules are included in the analysis.

## Materials and Methods

### Participants

In this study, data of four previous studies were combined (Boyen et al., 2013; Langers, 2014; Amaral and Langers, 2015; Koops et al., 2020a). All these studies were performed at the University Medical Center Groningen between 2012 and 2020. Data were available from 240 participants. In 21 scans, the cerebellum was only partially within the field of view. In 16 scans, an artifact was present in the cerebellum. These scans were excluded. This resulted in the inclusion of 203 participants, of whom 94 had tinnitus and 109 did not. Except for potential tinnitus and/or hearing loss, participants did not have a neurological or psychiatric diagnosis.

Hearing thresholds of all participants were determined with pure tone audiometry, performed in a sound attenuating booth. The thresholds were assessed using at least six different octave frequencies (0.25–8 kHz) for both ears separately. Mean hearing loss (PTA) was defined by the mean auditory thresholds at 2, 4 and 8 kHz of both ears. Additionally, tinnitus participants completed the Tinnitus Handicap Inventory (THI) to assess tinnitus handicap (Newman et al., 1996). This questionnaire consists of 25 questions assessing the functional (twelve items), emotional (eight items), and catastrophic reactions to tinnitus (five items). Total THI scores range from 0 to 100, in which 0 corresponds to no or light tinnitus burden and 100 to catastrophic tinnitus burden.

All studies were approved by the Research Ethical Board of the University Medical Center Groningen and performed in accordance with all relevant regulations. Written informed consent was obtained from all participants. The re-use of the data for this study was approved as well.

### MRI Data Acquisition

MRI scanning was performed on a 3.0T Philips Intera MRI scanner (Best, Netherlands) at the Neuroimaging Center (NiC) in Groningen. Whole brain T1 weighted anatomical images were acquired with a voxel size of 1 mm × 1 mm × 1 mm. The specifics on the acquisition parameters per subset of scans are reported in Table 1.

**TABLE 1:** MRI data acquisition parameters.

Study	Boyen et al. (2013) (*n* = 53)	Langers (2014) (*n* = 31)	Amaral and Langers (2015) (*n* = 36)	Koops et al. (2020a) (*n* = 83)
SENSE head coil	8-Channel	8-Channel	8-Channel	32-Channel
Number of slices	170	144	170	160
Acquisition duration (s)	251	160	251	608.5
Repetition time (ms)	9	9	9	10.4
Echo time (ms)	3.5	3.6	3.6	5.7
Flip-angle (°)	8	8	8	8
Matrix	256 × 256	192 × 192	256 × 256	256 × 256

### MRI Data Processing

**FIGURE 2:**
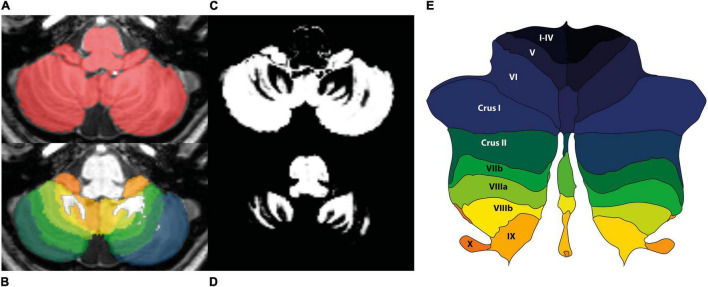
Sample images from one of the participants obtained with the SUIT pipeline. **(A)** Hand-corrected isolation map of the cerebellum in native space. **(B)** Cerebellum overlayed with lobular map in native space. **(C)** Tissue probability map gray matter in native space. **(D)** Tissue probability map white matter in native space. **(E)** Probabilistic lobular atlas of the regions of interest on a flatmap of the cerebellum. The colors correspond to the colors in panel **(B)**.

MRI data were analyzed with Statistical Parametric Mapping software (SPM12, FIL Wellcome Trust Center for Neuroimaging, London, United Kingdom), running in Matlab R2021a (Natick, MA, United States: The MathWorks Inc.). The data processing was performed using the spatially unbiased infratentorial template (SUIT) toolbox (Diedrichsen, 2006), which is optimized for cerebellar analysis. The authors were blinded to the tinnitus condition. All images were visually checked to ensure acceptable image quality, after which they were brought into LPI-orientation. The origin of the images was set on the anterior commissure. The cerebellum and brainstem were isolated, and the isolation maps were visually inspected and hand-corrected in MRIcroGL^1^, so that they only included infra-tentorial structures (Figure 2). Moreover, tissue probability maps of cerebellar gray and white matter were obtained. The segmentation maps were normalized and resliced to the SUIT-template using the Diffeomorphic Anatomical Registration Through Exponentiated Lie Algebra (DARTEL) algorithm. During spatial normalization, individual scans are warped to match the template. The resulting spatially normalized gray and white matter values were multiplied by its relative volume before and after spatial normalization (Jacobian determinant). This process of modulation ensures that the total amount of gray and white matter in a specific part of an individual’s brain is the same before and after spatial normalization. For that reason, when modulated spatially normalized images are used, VBM allows for comparison of the absolute volume of gray or white matter structures (Mechelli et al., 2005).

To obtain the Total Intracranial Volume (TIV) for each participant, the MRI scans were additionally processed using CAT12, a toolbox focused on structural images and the quantification of macroscopic structures in the brain. When TIV is used as a covariate, it takes individual differences in brain volume into account in the subsequent analyses. First, all T1 images were spatially normalized to the Montreal Neurologic Institute (MNI) 152 space using the high dimensional Diffeomorphic Anatomical Registration Through Exponentiated Lie Algebra (DARTEL). Second, images were segmented into gray matter, white matter and cerebrospinal fluid. The TIV was then calculated by summation of the volume of gray matter, white matter and cerebrospinal fluid.

The regions of interest (ROIs) were the bilateral cerebellar tonsils (lobules IX) and (P)FL-complexes (lobules X). Supplementary ROIs were the bilateral cerebellar lobules I-IV, V, VI, VIIb, VIIIa, and VIIIb, the bilateral crus I and II, the vermis of VI, crus 1, crus 2, VIIb, VIIIa, VIIIb, IX, and X, and the bilateral dentate, interposed and fastigial nuclei. Total cerebellar cortical GMV was obtained by summing up all bilateral cerebellar lobules. For each ROI, the GMV was extracted per participant.

### Statistical Analysis

All statistical analyzes were performed in R (v4.0.1) software. Statistical significance was established at *p* < 0.05, and Bonferroni-corrections were applied to correct for multiple comparisons when necessary. Differences in participant characteristics between the tinnitus and non-tinnitus groups were assessed with Mann-Whitney *U* tests. Differences in ROI GMVs between tinnitus participants and controls were assessed with t-tests or Mann-Whitney *U* tests, depending on normality. Normality was determined by the Shapiro-Wilk test. Next, a penalized ridge regression analysis with the outcome variable THI score was carried out within tinnitus participants. In this analysis, the variables tonsil GMV, (P)FL-complex GMV, total cerebellar cortex GMV, age, sex, PTA, TIV, and study that the data originated from were included. Ridge linear regression was applied to overcome the issue of multicollinearity in multiple linear regression, as tested by the variance inflation factor (VIF) (Duzan and Shariff, 2015). To include the categorical variable “original study,” it was coded as dummy variables, with the study of Boyen et al. (2013) as reference. The independent variables were centered and standardized, and the ridge parameter was chosen automatically using the method of Cule and De Iorio (2013). The R-packages {ridge} (Cule et al., 2021) and {lmridge} (Imdad and Aslam, 2021) were used for the analysis. Last, a Spearman’s partial correlation analysis was carried out with (P)FL-complex GMV and PTA, corrected for age, TIV, and original study.

## Results

### Participant Characteristics

**FIGURE 3:**
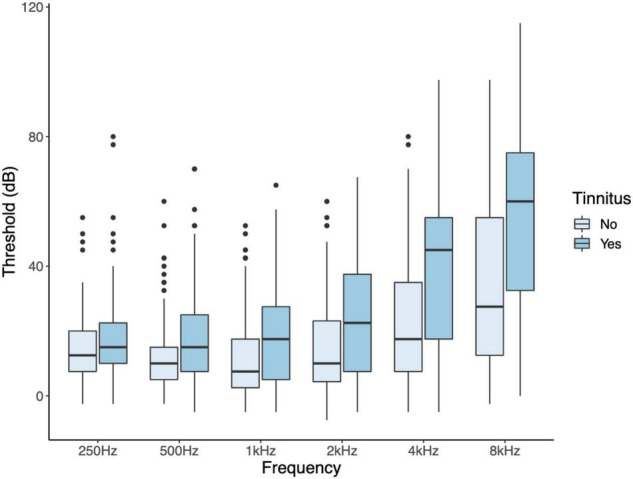
Audiograms of participants with and without tinnitus. Hearing thresholds of the frequencies 0.5, 1, 2, 4, and 8 kHz were significantly higher in tinnitus participants compared to the control group after correcting for multiple comparisons.

Since tinnitus laterality data was lacking and participants had symmetrical hearing, as evidenced by strong correlations between hearing thresholds of the left and right ear, the mean threshold of both ears was used. Participants with tinnitus were significantly older than participants without tinnitus, and their average hearing thresholds were higher (age: *W* = 4,139, *p* = 0.018; hearing threshold: *W* = 3,095, *p* ≤ 0.001; Table 2). The mean audiogram per group is shown in Figure 3. Hearing thresholds of the frequencies 0.5, 1, 2, 4, and 8 kHz were significantly higher in tinnitus participants compared to the control group after correcting for multiple comparisons (0.5 kHz: *W* = 3,750, *p* = 0.002; 1 kHz: *W* = 3,884, *p* = 0.006; 2 kHz: *W*, *p* = 0.001; 4 kHz: *W* = 2,953, *p* < 0.001; 8 kHz: *W* = 3,219, *p* < 0.001). The hearing threshold at 250 Hz did not differ between both groups. An increase in age was associated with an increase in PTA [r_s_(199) = 0.701, *p* < 0.001]. After correcting for multiple comparisons, no associations were present for age and THI, neither for PTA and THI.

**TABLE 2:** Participant characteristics.

Characteristics	Participants			*p*-value
	All (*n* = 203)	Tinnitus (*n* = 94)	No tinnitus (*n* = 109)	
Age	53 (18–84 years)	55 (19–76 years)	50 (18–84 years)	0.018
Sex (male)	113 (56%)	60 (64%)	53 (49%)	
Hearing thresholds				
PTA (2, 4, and 8 kHz)	31 (−3 to 88)	43 (-3 to 88)	19 (-3 to 77)	<0.001
250 Hz	13 (−3 to 80)	15 (-3 to 80)	13 (-3 to 55)	0.009^*D*^
500 Hz	13 (-5 to 70)	15 (-5 to 70)	10 (-3 to 60)	0.002^*D*^
1 kHz	10 (-5 to 65)	18 (-5 to 65)	8 (-5 to 53)	0.006^*D*^
2 kHz	18 (-8 to 68)	23 (-5 to 68)	10 (−8 to 60)	0.001^*D*^
4 kHz	28 (-5 to 98)	45 (-5 to 98)	18 (-5 to 80)	<0.001^*D*^
8 kHz	40 (-3 to 115)	60 (0 to 115)	28 (-3 to 98)	<0.001^*D*^
THI score	35 (4–82)	35 (4–82)		

*Data is presented as median (range) or n (%). p-values are based on Mann-Whitney-U tests to determine differences between tinnitus and non-tinnitus participants. ^*D*^Threshold for significance is Bonferroni corrected; p < 0.05/6 = 0.008.*

### Voxel Based Morphometry

Since tinnitus laterality data are not available for all included studies, GMV of the cerebellar lobules are presented as the mean of the left and right sides. No differences in GMV of the included cerebellar ROIs were identified between participants with or without tinnitus (Table 3).

**TABLE 3:** Differences in the gray matter volume (mm^3^) of multiple regions of interest between tinnitus and non-tinnitus participants.

Region of interest	Gray matter volume (mm^3^)		Test	*p*-value*
	Tinnitus	No tinnitus		
Lobules I–IV	3,376 ± 471	3,421 ± 433	*t*-test	0.487
Lobule V	4,130 ± 554	4,085 ± 530	*t*-test	0.557
Lobule VI	8,831 (2,905)	8,629 ± 1,135	Mann-Whitney *U*	0.69
Vermis VI	1,882 ± 261	1,860 ± 244	*t*-test	0.558
Crus I	13,153 ± 1,785	13,087 ± 1,698	*t*-test	0.788
Vermis crus I	19 ± 5	19 ± 5	*t*-test	0.995
Crus II	9,544 ± 1,366	9,448 ± 1,176	*t*-test	0.595
Vermis crus II	403 ± 59	399 ± 54	*t*-test	0.613
Lobule VIIb	4,930 ± 720	4,894 ± 614	*t*-test	0.706
Vermis VIIb	174 ± 26	176 ± 27	*t*-test	0.534
Lobule VIIIa	4,908 ± 712	4,864 ± 604	*t*-test	0.637
Vermis VIIIa	1,127 ± 161	1,127 ± 161	*t*-test	0.986
Lobule VIIIb	4,168 ± 572	4,088 ± 517	*t*-test	0.299
Vermis VIIIb	579 ± 87	572 ± 75	*t*-test	0.56
Lobule IX/tonsil	3,369 ± 504	3,317 ± 459	*t*-test	0.44
Vermis IX	698 ± 103	700 ± 98	*t*-test	0.902
Lobule X/(P)FL-complex	697 ± 97	681 ± 89	*t*-test	0.215
Vermis X	369 ± 72	365 ± 64	*t*-test	0.689
Dentate nucleus	1,563 ± 246	1,539 ± 232	*t*-test	0.473
Interposed nucleus	214 ± 37	208 ± 33	*t*-test	0.257
Fastigial nucleus	39 ± 8	38 ± 7	*t*-test	0.788

Data are presented as mean ± SD or median (interquartile range), depending on normality. *p-values < 0.05/21 = 0.0024 are considered significant.

To study the association between THI score and tonsil GMV, (P)FL-complex GMV, total cerebellar cortex GMV, age, sex, PTA, TIV, and the study the data originated from, a multiple linear regression was run. Multiple predictors were correlated with each other, which raised the issue of multicollinearity (the highest VIF was 23.2). We considered this to be data multicollinearity. Therefore, the analysis required the use of a penalized ridge linear regression model. The automatically chosen ridge penalty was 1.615. This reduced the VIF to 0.1 or lower for all variables. *R*^2^ was merely 0.024, with a *p*-value of 0.275 for the entire model. Only age was associated with THI score. No association was present between THI score and tonsil GMV, (P)FL-complex GMV, and total cerebellar cortical GMV (Table 4).

**TABLE 4:** Ridge linear regression on outcome variable THI score.

Variable	Regression coefficient	Scaled coefficient	Standard error (scaled)	*t*-Value (scaled)	*p*-Value
Tonsil GMV	–0.000	–0.802	4.833	0.166	0.869
(P)FL-complex GMV	–0.006	–5.536	5.222	–1.060	0.292
Total cerebellar cortical GMV	0.000	–0.069	4.485	–0.015	0.988
Age	–0.136	–15.199	6.911	–2.199	0.030*
Sex	0.170	0.788	7.167	0.110	0.913
PTA	–0.037	–7.458	6.235	–1.196	0.235
TIV	–0.004	–5.223	6.712	–0.778	0.439
Original study					
Langers (2014)	2.684	10.664	6.089	1.751	0.083
Amaral and Langers (2015)	–0.024	–0.084	6.841	–0.012	0.990
Koops et al. (2020a)	1.396	6.267	7.348	0.853	0.396

The automatically chosen ridge penalty was 1.615. R^2^ was merely 0.024, with a p-value of 0.275 for the entire model. Only age was significantly associated with THI score. No association was present between THI score and tonsil GMV, (P)FL-complex GMV and total cerebellar cortical GMV. Significant variables are marked with an asterisk (*).

The question remains why TFI was correlated to the bilateral (P)FL-complex volumes in Mennink et al. (2018), and whether that correlation depended on potential confounders such as hearing thresholds or TIV. In the current study, only THI scores were available, but no TFI scores. Therefore, THI score was used as an equivalent. In the current study population, there was no correlation between the (P)FL-complex GMV and THI scores, nor between TIV and THI scores. However, (P)FL-complex GMV did correlate with TIV [r(201) = 0.423, *p* < 0.001] and PTA [r_s_(199) = 0.308, *p* < 0.001] after correcting the threshold of significance with the Bonferroni correction (*p* < 0.05/4 = 0.013). Particularly the correlation with PTA is interesting. However, this correlation disappeared when we performed a Spearman’s partial correlation analysis between (P)FL-complex GMV and PTA, which controls for the effect of age, TIV, and the original study. The disappearance was driven by controlling for the original study.

## Discussion

In this study, we investigated the association between cerebellar GMV and tinnitus presence and burden. There was no difference in GMV of any cerebellar lobule between participants with or without tinnitus. Moreover, tinnitus burden, as measured by THI score, was only weakly associated with age, but not with tonsil GMV, (P)FL-complex GMV, nor total cerebellar cortical GMV.

The absence of significant differences in (P)FL-complex GMV between tinnitus and non-tinnitus participants in this study is in accordance with our previous study (Mennink et al., 2018). In our current study population there was a significant difference in age between participants with and without tinnitus. Moreover, it has been shown that total GMV declines with age (MacDonald and Pike, 2021). However, it seems unlikely that the minor mismatch in age between the tinnitus and non-tinnitus subjects is responsible for the lack of a tinnitus-related difference in this VBM analysis. Only one other study investigated cerebellar volume in subjective tinnitus (Sahin et al., 2020). Similarly, they reported no significant difference in the total flocculonodular lobe volume between tinnitus and non-tinnitus participants. In other anatomical regions of the cerebellum, we did not find any differences between tinnitus and non-tinnitus participants either. Sahin et al. (2020) reported a significantly smaller lobule IV, lobule V, and total central lobule, as well as a smaller quantity of total cerebellar white matter. Unfortunately, they did not correct for multiple comparisons. If they had done so, only the difference in lobule V volume would remain significant. In conclusion, there is only very little evidence for cerebellar GMV differences between people with and without tinnitus.

Our previous study showed a correlation between the Tinnitus Functional Index (TFI) score and the bilateral (P)FL-complex volumes, implying that patients with larger (P)FL-complexes suffered more from their tinnitus and vice versa (Mennink et al., 2018). Nevertheless, the current study did not find an association between self-reported tinnitus severity and (P)FL-complex GMV. The question arises why the results in both studies differ. Our previous study did not have access to audiometric data or TIV. Still, the absence of these potential confounders in the previous study is not enough to account for the relation between TFI score and (P)FL-complex volume. In the current study, this is evidenced by the lack of correlation between THI and (P)FL-complex GMV, in which was not accounted for hearing thresholds and TIV as well. Moreover, although hearing thresholds were not available in the previous study, most patients had substantial hearing loss or were completely deaf after tumor debulking. Thus, it is highly unlikely that the correlation between TFI score and (P)FL-complex volume in the previous study was based on PTA. Last, (P)FL-complex GMV did correlate with TIV, but TIV was not related to THI score in the ridge regression. In conclusion, it is not probable that the difference between both studies regarding an association between tinnitus severity and (P)FL-complex volume is based solely on the (lack of) inclusion of PTA and/or TIV.

Multiple possible explanations remain for the discrepancy of results between both studies. For instance, in the current study, the GMV was extracted, whereas, Mennink et al. (2018) reported on the entire volume of the (P)FL-complex. Moreover, the MRI-scans that were studied in Mennink et al. (2018) were T2-weighted images acquired by an 1.5T MRI-scanner, while the current study used T1-weighted images which were acquired by an 3.0T MRI-scanner. Thus, both studies used different MRI protocols, which could influence the results. Additionally, both studies used different tools to assess tinnitus severity. The THI score reflects tinnitus handicap severity, and the TFI score the negative impact of tinnitus on daily functioning. The THI and TFI show moderate agreement (Boecking et al., 2021; Santacruz et al., 2021). Since the agreement between both questionnaires is not perfect, it cannot be ruled out that the use of THI instead of TFI can (partly) explain the discrepancy between both studies. However, since the agreement is moderate, the THI score is likely sufficient to detect an association if it were present.

If we assume that the THI score would have detected an association between tinnitus severity and (P)FL-complex GMV if it existed, the explanation may be a difference in participant characteristics. In our previous work by Mennink et al. (2018), patients suffered from tinnitus after removal of a CPA tumor, whereas the current study focused on tinnitus participants who were otherwise healthy and did not have cranial surgery. Vestibular schwannomas account for 80–90% of CPA-tumors, and more than 95% is unilateral. The typical patient presents with asymmetric hearing loss (>90%), unilateral tinnitus (55%) and vestibular disturbances (61%) (Carlson and Link, 2021). Apart from damage to the nerve itself, large tumors compress neighboring structures such as the brainstem and cerebellum. Our initial hypothesis that (P)FL-complex volume is related to tinnitus severity, as measured by the THI questionnaire, is based on the assumption that the volume of a cerebellar area is related to its functionality. In traumatic brain injury, it has been shown that affected regional volumes were related to the severity of injury and the loss of function (Spitz et al., 2013). Thus, cerebellar compression due to a CPA tumor or manipulation during surgery could similarly lead to an altered function of the (P)FL-complex. This is evidenced by the fact that large CPA tumors cause extensive oculomotor abnormalities, since the FL is primarily involved in eye movement control (Nedzelski, 1983). Moreover, this would also explain why gaze-modulated tinnitus, in which gaze deviations modulate perceptual tinnitus characteristics such as loudness, is primarily identified in patients who have undergone CPA tumor removal (Baguley et al., 2006; van Gendt et al., 2012; Matsuno et al., 2016; Beh et al., 2017; Mennink et al., 2018). At the time that the studies, which were included in the current study, were performed, subjects completed several tinnitus-related questionnaires. These questionnaires did not evaluate the possible presence of gaze-modulated tinnitus. However, gaze-modulated tinnitus has not been not reported in patients without a neurological disease such as the current study population. This suggests that, because of the damage caused by a CPA-tumor, the normal function of the FL in gaze-control has become associated with changes in perceptual tinnitus characteristics. Moreover, it suggests that atrophy of the (P)FL-complex, due to compression by the CPA-tumor or manipulation of the (P)FL-complex during surgery, could lead to an altered function and abnormal cross-modal interactions (Mennink et al., 2018). Possibly, the relation between cerebellar GMV and tinnitus only emerges if the damage to the (P)FL-complex is extensive enough, as is the case in CPA tumors and subsequent surgery. In tinnitus participants without a CPA tumor the activity of the PFL, and the functional connectivity between the auditory cortex and PFL, might be increased just like in animals (Chen et al., 2015). The increased activity may be altered by the damage of the PFL because of the CPA tumor. Thus, in patients with large tumors, the damage is more significant, and the function of the PFL in tinnitus (increased connectivity and activity) is deteriorated. This would lead to a relation between tinnitus severity and (P)FL-complex volume, in which a smaller (P)FL-complex leads to less severe tinnitus and vice versa, as observed in Mennink et al. (2018). On the other hand, the effect of a CPA-tumor and subsequent surgery on the ipsilateral hearing thresholds may explain the existence of a relation between TFI and (P)FL-volume. CPA tumor patients usually have substantial hearing loss or are completely deaf in the affected ear after surgery (Maw et al., 2003; van Gendt et al., 2012; Xia et al., 2017). Although Mennink et al. (2018) lacked audiometric data, most patients reported that they were extremely bothered by hearing loss after surgery. Therefore, it is probable that most patients had at least substantial hearing loss in the affected ear as well. However, in the current study, participants had moderate, symmetrical hearing loss at most. So, the relation between (P)FL-complex and tinnitus severity may only emerge when hearing loss in the ipsilateral ear is at least substantial, or when hearing thresholds between both ears are asymmetrical. Last, patients with CPA-tumors often suffer of unilateral tinnitus, as was the case in Mennink et al. (2018). It is probable that most participants in the current study suffered of bilateral tinnitus, although we do not have data on the laterality of tinnitus. Thus, it may also be the case that the relation between (P)FL-complex volume and tinnitus severity is only present in unilateral tinnitus. Nevertheless, the different study populations may explain the discrepancy between both studies.

Alternatively, potential tinnitus-related changes in (P)FL-complex GMV in otherwise healthy participants may be too small to detect with the current methodology. Previous work has shown that tinnitus burden can vary over time and that there is a complex relation between tinnitus burden and sleep quality, stress, anxiety, and depression (Neff et al., 2021). It is unlikely that the structural changes resulting from these factors are as large as the damage done by a CPA tumor or cranial surgery. Therefore, small tinnitus-related alterations in the GMV of the cerebellar lobules, as expected in an otherwise healthy population, may remain undetected by this type of voxel-based analysis.

Previous publications suggest that the PFL can modulate tinnitus (Mennink et al., 2020), and small differences in the structure or the function of the (P)FL may play a role in tinnitus. Since morphometry studies cannot extract neural activity, future work should focus on including functional and diffusion scans of the cerebellum to unravel its role in the pathophysiology of tinnitus in humans.

### Limitations

A limitation of this study is the absence of laterality data. Animal studies showed increased activity of the PFL after unilateral noise trauma, specifically ipsilateral to the trauma ear (Brozoski et al., 2007). Similarly, intervention within the ipsilateral PFL, through pharmacological blockade or activation, or ablation of the ipsilateral PFL, modulated tinnitus (Bauer et al., 2013a,b; Brozoski et al., 2013). Thus, these studies clearly show the importance of laterality. Moreover, in patients with CPA tumors the tinnitus is often lateralized to the side of the tumor (van Gendt et al., 2012). Whereas in most cases tinnitus is bilateral, the lack of laterality data could have influenced the results (Levine, 2013).

Another limitation of this study is that the data used in this study, was collected with four different study protocols and consisted of slightly different study samples. The impact hereof was illustrated by the effect of adding the original study as a variable in the partial correlation analysis. Moreover, the current study measured the GMV of the whole (P)FL-complex, since the tight folding of the cerebellum prevents precise identification of the substructures of the PFL on a human structural MRI-scan. Complete unfolding of the cerebellum, with recognition of individual folia, requires a resolution that is typically not available in human imaging studies (Diedrichsen and Zotow, 2015).

## Conclusion

The current study showed no relation between the GMV of the (P)FL-complex or other cerebellar lobules and tinnitus presence or severity. So, in contrast to patients that underwent CPA tumor removal, there is no clear relationship between (P)FL-complex GMV and tinnitus in otherwise healthy participants. These findings may indicate that this relation only exists when the (P)FL-complex is damaged, for instance by a CPA tumor. Alternatively, it is possible that the differences in (P)FL-complex volumes are too small to detect with a VBM study.

## Data Availability Statement

The data analyzed in this study is subject to the following licenses/restrictions: Due to privacy regulations, the original MRI-scans are not available. However, numerical data and code may be provided to interested researchers upon request to the corresponding author, after clearance by the research ethical board. Requests to access these datasets should be directed to LM, l.m.mennink@umcg.nl.

## Ethics Statement

The studies involving human participants were reviewed and approved by the Research Ethical Board of the University Medical Center Groningen. The patients/participants provided their written informed consent to participate in this study.

## Author Contributions

LM, EK, PD, and JD designed the experiment. LM and EK analyzed the data. LM wrote the draft of the manuscript. All authors discussed the results, commented on the manuscript, and approved the submitted version.

## Conflict of Interest

The authors declare that the research was conducted in the absence of any commercial or financial relationships that could be construed as a potential conflict of interest.

## Publisher’s Note

All claims expressed in this article are solely those of the authors and do not necessarily represent those of their affiliated organizations, or those of the publisher, the editors and the reviewers. Any product that may be evaluated in this article, or claim that may be made by its manufacturer, is not guaranteed or endorsed by the publisher.

## Footnotes

1
https://www.nitrc.org/projects/mricrogl

## Abbreviations

CPA: cerebellopontine angle
FL: flocculus
GMV: gray matter volume
PFL: paraflocculus
PTA: pure tone average
ROI: region of interest
TFI: Tinnitus Functional Index
THI: Tinnitus Handicap Inventory
TIV: total intracranial volume
VBM: voxel-based morphometry
VIF: variance inflation factor.
